# From Knowledge to Action: Fostering Advocacy Skills for Planetary Health in Physical Therapy

**DOI:** 10.1093/ptj/pzae130

**Published:** 2024-09-06

**Authors:** Emma Swärdh, Filip Maric

**Affiliations:** Division of Physiotherapy, Department of Neurobiology, Health Care Sciences and Society, Karolinska Institutet, Huddinge, Sweden; Department of Health and Care Sciences, UiT the Arctic University of Norway, Tromsø, Norway

## Introduction

Today’s world is increasingly marked by complex threats to health posed by biodiversity loss, climate change, social inequality, and surging geopolitical conflicts, with children, the elderly, and people with ill health facing increased vulnerability.^1^^,^^2^ To address these intersecting challenges effectively, health care professionals must develop new knowledge and skills through higher education and continuous professional development. Following the United Nations Agenda 2030 and United Nations Educational, Scientific, and Cultural Organization’s competencies for sustainable development,^3^ there is also an ethical imperative to address the multifaceted health consequences of biodiversity loss and climate change, facilitating transformative change for healthy and sustainable futures. Planetary health advocacy represents a critical skill and mode of practice for health professionals in this regard, grounded on a novel, integrated understanding of health, environment, and society.^4^ We present a call for action to equip future and current physical therapists with the necessary knowledge and skills for planetary health advocacy as an ethical health professional practice.

## Rationale for Planetary Health

Health professional organizations, communities, and student-led networks all over the world have made efforts to recognize planetary health advocacy as an integrated part of their professions in different policies, statements, and curricula proposals.^5–8^ The 2021 São Paulo Declaration on Planetary Health emphasized the need for support and action from all health care professionals, highlighting the urgency to use their positions as trusted members of society to advocate for planetary health.^9^ World Physiotherapy has also issued its own climate change and health position statement, encouraging its member organizations to advocate and collaborate for climate change mitigation and adaptation. ^10^ Member organizations, including the American Physical Therapy Association,^11^ the Chartered Society of Physiotherapy,^12^ the Swedish Association of Physiotherapists,^13^ and others, are indeed, increasingly issuing their corresponding statements on environmental sustainability, stewardship, and the health effects of environmental degradation, followed by initial actions at different organizational levels.

Within its education framework, World Physiotherapy has further already embedded advocacy within several domains of competence, including ethical and professional practice, interprofessional teamwork, and leadership and management.^14^ Though there is no universally accepted definition of health advocacy, it is often understood to encompass advocating for health equity and doing so with or for an individual, group, community, or population.^15^ Where patient education mainly takes place at an individual level, advocacy is directed at meso or macro levels, whether this is by siding with patients, communities, or initiatives to bring about change in their local context or working toward systems change by involving government or other large-scale lobbying building on a population-health perspective.^16^ Health advocacy thus requires collaboration with various sectors, such as community agencies, social services, patient organizations, or educational institutions, and it will consequently differ according to the specific cause and context in which it is directed.

Taking together the recent calls for climate action in the health professions, and the extant understanding of health advocacy, then, provides a solid foundation for physical therapists’ engagement in planetary health advocacy. The additional opportunity provided by planetary health is to broaden the scope of our advocacy practice to integrate strategies for the social and ecological determinants of health at both meso or macro levels.^17^

## Advocacy for Planetary Health

Patient education for planetary health is often thought to involve education about health behaviors that consider co-benefits for health and the environment, including examples like adopting active lifestyles and transport choices, eco-conscious consumers, and dietary choices. Advocacy, then, has to be directed at community, institutional, social, economic, or political conditions that make it possible for people to adopt corresponding health behaviors.

Physical therapists might begin by advocating for socially just and eco-friendly approaches within health care and physical therapist service delivery while setting an example in their professional behaviors and clinical practices, such as reducing waste, recycling, and promoting zero-emission transport and plant-based food. Advocating for institutional change might also include leading initiatives to transition to renewable electricity, creating waste reduction policies, energy conservation measures, and circular economy principles within the health care institution.^18–20^

Beyond one’s immediate practice environment, but still within the health sector, challenging existing national health care systems and advocating for decarbonization of health care, as well as for innovative health care structures that prioritize sustainability and planetary health, constitute critical advocacy actions.^17^ Other actions might include crafting evidence-informed messages about planetary health directed at the general public, or specific target groups and communication channels.^21^ These messages should inspire collective action, and be adapted to each country’s policies, systems, laws, and regulations. Resonant actions include writing insightful scientific commentaries and debate articles to raise collegial and/or public awareness, hosting and participating in podcasts, or disseminating information to the public through seminars, workshops, and social media.

Physical therapists also have an opportunity to influence health-related policies outside health care to become more sustainable and climate-friendly. For example, policies related to landscape design, urban planning, and transport infrastructure that facilitate use of active transportation,^22^ access to green spaces, and exposure to nature-based health and therapy^23^ are strongly encouraged. Collaboration with local communities and networks will be paramount to address health disparities and environmental concerns through initiatives that tackle both public health and climate-related challenges. Lobbying together with environmental organizations for national policies that prioritize biodiversity conservation is another way of using expertise on the intersection of biodiversity loss and health. Given that social and environmental integrity are fundamental to human health, planetary health advocacy can also encompass actively supporting or initiating social and environmental campaigns, petitions, and even protests, either directly in or at varying distances from relevant environments.

## Cultivating Advocacy Competencies in Physical Therapy

Many health care professionals are already dedicated to social and environmental concerns in their practice and are willing to advocate for planetary health. There is, however, a sense of ambiguity regarding the timing and approach to address these issues, potentially related to barriers such as limited knowledge, time, and resources or lacking support from colleagues.^24^^,^^25^ Thus, providing efficient continuing professional education is urgent. Developing courses within global professional organizations, such as the Environmental Physiotherapy Association^26^ or creating national platforms, such as the American Physical Therapy Association Environmental Physical Therapy Catalyst Group,^27^ Physiotherapy Declares in the United Kingdom,^28^ Health for Future movement in Germany,^29^ and others can serve as a starting point for networking within planetary health advocacy. The global student-led initiative Planetary Health Report Card can also facilitate positive change within physical therapist education by evaluating advocacy efforts associated with planetary health within institutions and faculty.^30^ Within undergraduate or graduate health professional education, some educators may still feel hesitant to discuss planetary health advocacy due to overcrowded curriculum, political discourse, or low belief in making a difference.^31^ However, the responsibility of higher education today is to prepare students for new challenges that need to be met with new responses, precisely including advocacy as an engagement in health care professional practice for planetary health.

Thankfully, the tides are changing on this latter point as relevant frameworks and guidelines are providing growing support for implementation. A recent guidance for allied health professions in the United Kingdom identifies planetary health advocacy as 1 of 5 foundations of education for sustainable health care.^32^ The Framework for Planetary Health Education^33^ similarly emphasizes advocacy as a critical means to facilitate systemic change and drive the transition toward a more equitable future. To prepare current and future physical therapists and make planetary health advocacy an integral aspect of professional identity and practice, professional education must provide tools and guidance for physical therapists to act on health issues outside the familiar clinical context.

## Integrating Cognitive, Affective, and Psychomotor Aspects of Learning for Planetary Health

Developing competencies for effective planetary health advocacy in physical therapists requires a pedagogical approach that embraces both cognitive and affective, as well as psychomotor aspects of learning.^34^

### Understanding the Landscape–Cognitive

Before engaging in advocacy, physical therapists must learn about the fundamental dependence of human health on the natural environment, including the basics of climate science and biodiversity, and the ways that environmental degradation impacts health at different levels. Current and future physical therapists must be encouraged to explore interdisciplinary research as well as intersectoral policies and develop a thirst for collaboration across disciplinary boundaries. Such transdisciplinarity is essential to address advocacy efforts at various stakeholders and advance informed decision-making on climate-related health issues.

### Fostering Empathy and Connection–Affective

Effective advocacy necessitates an emotional connection beyond facts and figures. Physical therapists must be given the opportunity to immerse themselves in authentic narratives grounded in people’s lived experiences. Exploring and reflecting on, for example, how a patient has illness during an extreme weather event or how air pollution has affected a child’s health might foster emotions such as empathy and compassion but also a sense of alarm that motivates and prepares physical therapists to go outside the comfort zone of their usual clinical practice and inspire action. However, greater awareness of environmental issues and their implications for health can also trigger worry, grief, and anxiety, leading to action-disabling paralysis. An important solution to this might be emphasizing and guiding through the many ways that health professional can be active, with advocacy presenting a critical avenue for practice.

### Committing to Action—Psychomotor

The final step in effective advocacy education involves taking concrete actions to drive change. Just like with other clinical practice education, education for advocacy must also include opportunities for learners to practice advocacy, adapting their respective messages to different audiences and influencing various stakeholders. Learners could, for example, actively engage in institutional advocacy to increase planetary health awareness and education, promoting whole-institution approaches and accountability within higher education. Through such practice, learners should be guided in the development of their communication skills, including, for example, storytelling, social marketing strategies, and diffusion of innovations, to make their messages more accessible and meaningful to diverse audiences. Co-creation with students and colleagues from other disciplines, as well as with educators, should be encouraged to foster interdisciplinary and intergenerational collaboration in planetary health advocacy, increase creativity, and connect with broader audiences on a deeper level.

## The Path Forward

As we navigate the evolving landscape of health care, it becomes increasingly evident that planetary health advocacy is a timely expansion to physical therapists increasing engagement in health advocacy. The path forward involves not only recognizing the interconnectedness of environmental and human health but also actively engaging in initiatives that promote sustainability, equity, and resilience for both people and the planet. To ensure effective engagement in planetary health advocacy, physical therapists need educational opportunities to develop the necessary cognitive knowledge, affective attitudes, and psychomotor skills needed to address the complex challenges of our time and become the change-makers our world so urgently needs.

## References

[1] Rocque RJ , Beaudoin C, Ndjaboue R et al. Health effects of climate change: an overview of systematic reviews. BMJ Open. 2021;11:e046333. 10.1136/bmjopen-2020-046333. PMC8191619 34108165

[2] Robinson JM , Breed AC, Camargo A, Redvers N, Breed MF. Biodiversity and human health: a scoping review and examples of underrepresented linkages. Environ Res. 2024;246:118115. 10.1016/j.envres.2024.118115. 38199470

[3] Rieckmann M . Learning to transform the world: key competencies in education for sustainable development. In: Leicht A, Heiss J, Byun W.J, eds. Issues and Trends in Education for Sustainable Development. Paris, France: UNESCO Publishing; 2018:39–59. https://unesdoc.unesco.org/ark:/48223/pf0000261802.

[4] Barna S , Maric F, Simons J, Kumar S, Blankestijn PJ. Education for the anthropocene: planetary health, sustainable health care, and the health workforce. Med Teach. 2020;42:1091–1096. 10.1080/0142159X.2020.1798914. 32805141

[5] International Federation of Medical Students’ Associations . IFMSA Policy Document Climate Change. Copenhagen, Denmark: IMSA International Secretariat; 2023. Accessed February 2, 2024. https://ifmsa.org/wp-content/uploads/2023/05/IFMSA-Policy-Document-on-Climate-Change.docx.pdf.

[6] American Nursing Association . Nurses’ role in addressing global climate change, climate justice, and health. Position statements. 2023. Accesses February 2, 2024. https://www.nursingworld.org/practice-policy/nursing-excellence/official-position-statements/id/climate-change/.

[7] Atwoli L , Baqui AH, Benfield T et al. Call for emergency action to limit global temperature increases, restore biodiversity, and protect health: wealthy nations must do much more, much faster. Phys Ther. 2021;101:pzab193. 10.1093/ptj/pzab193. PMC8459882 34486055

[8] Li LSK , Fryer CE, Chi L, Boucaut R. Physiotherapy and planetary health: a scoping review. Eur J Phys. 2024;1–11. 10.1080/21679169.2024.2323729.

[9] Myers SS , Pivor JI, Saraiva AM. The São Paulo declaration on planetary health. Lancet. 2021;398:1299. 10.1016/S0140-6736(21)02181-4. PMC8492019 34624245

[10] World Physiotherapy . Policy statement. Climate change and health. 2023. Accessed February 2, 2024. https://world.physio/policy/ps-climate-change-and-health.

[11] American Physical Therapy Association . Support of Environmentally Responsible Practice by the American Physical Therapy Association. Alexandria, VA. Updated August 13, 2020. Accessed May 22, 2024. https://www.apta.org/siteassets/pdfs/policies/support-environmentally-responsible-practice.pdf.

[12] Chartered Society of Physiotherapy . CSP Declares Climate and Nature Emergency. April 6, 2022. Accessed May 22, 2024. https://www.csp.org.uk/news/2022-04-06-csp-declares-climate-nature-emergency.

[13] Swedish Association of Physiotherapists (Fysioterapeuterna) . Hållbarhet. Fysioterapeuterna. Accessed May 22, 2024. https://www.fysioterapeuterna.se/om-oss/om-oss/hallbarhet/.

[14] World Physiotherapy . Physiotherapist education framework. 2021. Accessed February 2, 2024. https://world.physio/sites/default/files/2021-07/Physiotherapist-education-framework-FINAL.pdf.

[15] Hubinette M , Dobson S, Scott I, Sherbino J. Health advocacy. Med Teach. 2017;39:128–135. 10.1080/0142159X.2017.1245853. 27866451

[16] Reid L . Ethics of advocacy. Rhode Island Med J. 2022;105:13–16 http://www.rimed.org/rimedicaljournal/2022/04/2022-04-13-ethics-reid.pdf. 35349613

[17] Howard C , MacNeill AJ, Hughes F et al. Learning to treat the climate emergency together: social tipping interventions by the health community. Lancet Planet Health. 2023;7:e251–e264. 10.1016/S2542-5196(23)00022-0. 36889866

[18] Duindam D . Transitioning to sustainable healthcare: decarbonising healthcare clinics, a literature review. Challenges. 2022;13:68. 10.3390/challe13020068.

[19] Maric F , Groven KS, Banerjee S, Michelsen TD. Essentials for sustainable physiotherapy: introducing environmental reasoning into physiotherapy clinical decision-making. Fysioterapeuten. 2021;4:54–58. https://www.fysioterapeuten.no/fagfellevurdert-sustainable-physiotherapy/essentials-for-sustainable-physiotherapy-introducing-environmental-reasoning-into-physiotherapy-clinical-decision-making/131740.

[20] Environmental Physiotherapy Association . The Sustainable Physiotherapy Clinic. Accessed February 6, 2024. https://environmentalphysio.com/practice/the-sustainable-physiotherapy-clinic/.

[21] Uppalapati S , Ansah P, Campbell E et al. A Global Review of Research on Effective Advocacy and Communication Strategies at the Intersection of Climate Change and Health. George Mason University; 2023. Accessed February 2, 2024: https://www.climatechangecommunication.org/wp-content/uploads/2023/11/Climate-and-Health-Literature-Review-Final.pdf.

[22] Toner A , Lewis JS, Stanhope J, Maric F. Prescribing active transport as a planetary health intervention – benefits, challenges and recommendations. Phys Ther Rev. 2021;26:159–167. 10.1080/10833196.2021.1876598.

[23] Stanhope J , Maric F, Rothmore P, Weinstein P. Physiotherapy and ecosystem services: improving the health of our patients, the population, and the environment. Physiother Theory Pract. 2023;39:227–240. 10.1080/09593985.2021.2015814. 34904927

[24] Kotcher J , Maibach E, Miller J et al. Views of health professionals on climate change and health: a multinational survey study. Lancet Planet Health. 2021;5:e316–e323. 10.1016/S2542-5196(21)00053-X. 33838130 PMC8099728

[25] Chi L , Boucaut R, Li LK, Fryer CE, Kumar S. Australian physiotherapists' knowledge and views on the relationship between climate change, health, and physiotherapy. Physiother Res Int. 2024, 2024;29:e2085. 10.1002/pri.2085. 38546164

[26] Environmental Physiotherapy Association. Accessed May 22, 2024.

[27] American Physical Therapy Association Academy of Leadership & Innovation . Environmental PT Catalyst Group. Accessed May 22, 2024. https://www.aptaali.org/page/CatalystGroups.

[28] Health Declares Climate and Ecological Emergency. Physiotherapy Declares. UK: Health Declares. Accessed May 22, 2024. https://healthdeclares.org/physiotherapydeclares/.

[29] Health for Future. Health for Future movement. Accessed May 22, 2024. https://healthforfuture.de/english/.

[30] Planetary Health Report Card. Planetary Health Report Card Initative. PHRC; 2023. Accessed May 22, 2024: https://phreportcard.org/.

[31] Brennan ME , Madden DL. The evolving call to action for including climate change and environmental sustainability themes in health professional education: a scoping review. J Clim Change Health. 2023;9:100200. 10.1016/j.joclim.2022.100200.

[32] Council of Deans of Health . Education for sustainable healthcare within UK pre-registration curricula for allied health professions. UK: Council of Deans of Health; 2023. Accessed February 2, 2024. https://www.councilofdeans.org.uk/2023/12/guidance-education-for-sustainable-healthcare/.

[33] Guzmán CAF , Aguirre AA, Astle B et al. A framework to guide planetary health education. Lancet Planet Health. 2021;5:e253–e255. 10.1016/S2542-5196(21)00110-8. 33894134

[34] Sipos Y , Battisti B, Grimm K. Achieving transformative sustainability learning: engaging head, hands and heart. Int J Sustain High Educ. 2008;9:68–86. 10.1108/14676370810842193.

